# Supplementary material for: Molecular approaches reveal weak sibship aggregation and a high dispersal propensity in a non‐native fish parasite
Source: Ecol Evol. 2021 May 6;11(11):6080–90. doi: 10.1002/ece3.7415 (PMC8207417; doi:10.1002/ece3.7415)
Supplement: Supplementary file 1 — Appendix S1‐S5 [file ECE3-11-6080-s001.docx]

**APPENDICES**

**Appendix S1.** As a part of preliminary work for a previous study (Loot et al., 2011), we genotyped 20 eggs (5 eggs per egg sac per female for 2 females) at several polymorphic microsatellite loci and found a maximum of 2 alleles per locus across eggs from the same female, clearly indicating that all eggs originate from the same unique father (unpublished data).

**Appendix S2.** Main characteristics of the 16 microsatellite markers used in *Tracheliastes polycolpus* and composition of the two PCR multiplexes. Discarded loci are in italics. He: expected heterozygosity; Ho: observed heterozygosity; Na: number of alleles.

| **PRIMER NAME** | **MULTIPLEX (and DYE)** | **PCR PRODUCT SIZE (min-max)** | **PRIMER LEFT SEQUENCE** | **PRIMER RIGHT SEQUENCE** | **MOTIF** | **He** | **Ho** | **Na** |
| --- | --- | --- | --- | --- | --- | --- | --- | --- |
| TRA5 | B (FAM) | 151-159 | CAGTGGGCAACAAAGGAAAT | AGTTTGGGGAAAACTTGGCT | ATTG | 0.130 | 0.131 | 4 |
| TRA6 | B (HEX) | 193-197 | AAAGGAAAGGCATTTGACCC | GGATGTGTCGAAAAAGCGAT | CCTT | 0.485 | 0.504 | 2 |
| *TRA12* | *A (FAM)* | *164-184* | *CTCAATCAACAAACAAAAGTTTC* | *GAAAAAGCTCAGACAAAAGTATGC* | *TATC* | *0.434* | *0.439* | *6* |
| TRA20 | B (FAM) | 195-197 | CAGGATGAGGATGAATTAGCG | CACGTTGTTTTTGATTCGGA | AG | 0.362 | 0.367 | 2 |
| TRA33 | B (ATTO550) | 188-196 | CAGTCGAAAGTGCGCAATAA | ATTTTTGAGGCGACTACGGA | AG | 0.669 | 0.678 | 5 |
| TRA42 | A (FAM) | 264-280 | CACAAAGTGCATGGAAATGG | GCGTCTCTGAAGGTGATTACG | GA | 0.640 | 0.663 | 5 |
| TRA44 | A (HEX) | 275-279 | CCTGAATTCGAATGAAAACCA | GGAGAATGTAATCGGAAAATCC | GA | 0.638 | 0.639 | 3 |
| TRA49 | B (HEX) | 288-294 | AGGACTTTCGGAGCTGATGA | CACACAGACAGACACAGTCACAA | GA | 0.511 | 0.505 | 5 |
| TRA53 | B (ATTO550) | 314-322 | CCCACAAAAATGCTTTGGTT | CATCTTTGTAAGACGAGTTTGCTG | TC | 0.572 | 0.566 | 5 |
| TRA58 | A (FAM) | 316-330 | TATCGCTGAGAAAGGCACTG | GACGTTTCTACCGGCACTTC | GA | 0.513 | 0.507 | 5 |
| TRA59 | A (HEX) | 330-336 | GGCAGCATCAAATTGTTTGT | CTCGAGACTTTACGGCCATC | TC | 0.479 | 0.497 | 4 |
| TRA62 | B (FAM) | 344-366 | CGGGAAGAGGACGAAAAGA | TCATTTCCGATGATGGCATA | TC | 0.691 | 0.686 | 6 |
| *TRA66* | *B (HEX)* | *351-361* | *TCCTGTATGCCCAAAACACA* | *TCATCAAATAATAAAATCCTTCTTTTCTC* | *AG* | *0.393* | *0.210* | *4* |
| TRA76 | A (ATTO550) | 115-139 | CAGCAACTGTAAGATATTAGCAAC | ACTCGCCGCTAAACACAAG | TA | 0.681 | 0.698 | 12 |
| TRA81 | A (FAM) | 111-115 | TGTCCATCTCCTTGAGTGGC | AGGCCTCGTTCCTTCGTATT | AG | 0.417 | 0.423 | 3 |
| TRA90 | A (ATTO550) | 189-199 | AGATGTCAAACCCTGGGATG | TTCCATAACCCAACAGGGAC | TC | 0.473 | 0.486 | 5 |

**Appendix S3.** Distribution of the number of full-sib members obtained in the 160 reconstructed full-sib families of *T. polycolpus*.

**Appendix S4.** Observed proportion of full-sibs sharing the same host within each site (Obs) compared to the series of expected proportions of full-sibs infecting the same host within each site under the null hypothesis (i.e., pairs of full-sibs are distributed randomly among hosts within each site) obtained after 10,000 permutations of the host matrix.

**Appendix S5.** Distribution of the family core location estimated for the reconstructed full-sib families of *T. Polycolpus* including more than five full-sibs.
